# Using the intervention mapping protocol to reduce European preschoolers’ sedentary behavior, an application to the ToyBox-Study

**DOI:** 10.1186/1479-5868-11-19

**Published:** 2014-02-19

**Authors:** Ellen De Decker, Marieke De Craemer, Ilse De Bourdeaudhuij, Vera Verbestel, Kristin Duvinage, Violeta Iotova, Evangelia Grammatikaki, Andreas Wildgruber, Theodora Mouratidou, Yannis Manios, Greet Cardon

**Affiliations:** 1 Department of Movement and Sports Sciences, Ghent University, Watersportlaan 2, 9000 Ghent, Belgium; 2 Division Metabolic and Nutritional Medicine, Dr. Von Hauner Children’s Hospital, Ludwig-Maximilians-University of Munich, Lindwurmstr.4, D-80337 München, Germany; 3 Clinic of Paeditric Endocrinology, UMHAT "St. Marina", Hr. Smirnenski Blvd, Varna, Bulgaria; 4 Department of Nutrition and Dietetics, Harokopio University, 70 El. Venizelou ave, 17671 Kallithea, Greece; 5 Staatsinstitut für Frühpädagogik (IFP), State Institute of Early Childhood Research, IFP, Winzererstr. 9, Eckgebäude Nord, 80797 München, Germany; 6 University of Zaragoza, GENUD Group, Edificio Cervantes, C/ Corona de Aragón 42, 50009 Zaragoza, Spain

**Keywords:** Kindergarten, Preschoolers, Sedentary behavior, Intervention Mapping Protocol

## Abstract

**Background:**

High levels of sedentary behavior are often measured in preschoolers, but only a few interventions have been developed to counteract this. Furthermore, detailed descriptions of interventions in preschoolers targeting different forms of sedentary behavior could not be located in the literature. The aim of the present paper was to describe the different steps of the Intervention Mapping Protocol used towards the development of an intervention component of the ToyBox-study focusing on decreasing preschoolers’ sedentary behavior. The ToyBox-study focuses on the prevention of overweight in 4- to 6-year-old children by implementing a multi-component kindergarten-based intervention with family involvement in six different European countries.

**Methods:**

Applying the Intervention Mapping Protocol, six different steps were systematically completed for the structured planning and development of the intervention. A literature search and results from focus groups with parents/caregivers and kindergarten teachers were used as a guide during the development of the intervention and the intervention materials.

**Results:**

The application of the different steps in the Intervention Mapping Protocol resulted in the creation of matrices of change objectives, followed by the selection of practical applications for five different intervention tools that could be used at the individual level of the preschool child, at the interpersonal level (i.e., parents/caregivers) and at the organizational level (i.e., kindergarten teachers). No cultural differences regarding preschoolers’ sedentary behavior were identified between the participating countries during the focus groups, so cultural and local adaptations of the intervention materials were not necessary to improve the adoption and implementation of the intervention.

**Conclusions:**

A systematic and evidence-based approach was used for the development of this kindergarten-based family-involved intervention targeting preschoolers, with the inclusion of parental involvement. The application of the Intervention Mapping Protocol may lead to the development of more effective interventions. The detailed intervention matrices that were developed as part of the ToyBox-study can be used by other researchers as an aid in order to avoid repetitive work for the design of similar interventions.

## Background

Sedentary behavior is often defined as activities involving sitting down [1]. Recently, the Sedentary Behavior Research Network suggested the use of a standardized definition of sedentary behavior. This definition describes that sedentary behavior includes activities that are characterized by an energy expenditure of ≤ 1.5 Metabolic equivalent of Task (MET), mostly during sitting or in a reclining position (e.g., watching television (TV), using the computer) [2]. Recent studies found that higher levels of sedentary behavior were associated with negative health outcomes, like less desirable cognitive and behavioral outcomes [3,4], and with a lower bone mineral content in children [5]. Furthermore, sedentary behavior (and in particular screen viewing behaviors) has been associated with overweight in children [6-8]. Consequently, different health-enhancing guidelines have been formulated that recommend limiting the length of time in sedentary behaviors in general [1,9-12], minimizing screen time including TV viewing and the use of other electronic media (e.g., DVD, computers, electronic games) to less than one to two hours per day in young children [13].

However, objective and subjective monitoring studies indicate that preschoolers (4 to 6 years) spend much of their time in sedentary activities [14-16]. Screen-based activities are generally included in preschoolers’ daily routine [16], with reports that indicated that children below the age of six years watched almost two hours of TV per day [17]. Furthermore, parental reports in the study of Cardon and De Bourdeaudhuij indicated that preschoolers between 4- and 5-years-old viewed TV or played on the computer for an average of 74 minutes on weekdays and 140 minutes on weekends [18]. High levels of sedentary behavior are observed at home as well as at organized out-of-home care (e.g., in preschools or child-care centers), with great variability’s of this behavior between centers [19,20]. Brown et al. for example reported that almost 89% of preschoolers’ time at preschool was spent in a sedentary way [21], while Temple et al. reported that preschoolers spent 39.5 minutes per hour in sedentary behavior in family child care [22]. Preschoolers are not only sedentary during the time they spend inside the classroom; high sedentary behavior levels were also objectively measured during preschool recess [23,24].

Although high levels of sedentary behavior are reported in different forms and settings, only a limited number of interventions focusing on decreasing this behavior has been conducted in preschoolers. Two review articles evaluated interventions focusing on decreasing screen time in children [25] and on limiting sedentary behavior [26]. The review of DeMattia et al. [26] included only one school-based intervention targeting preschoolers that executed a 7-session program with a weekly 20-minutes educational session in children between 2 and 5 years old [27]. Findings showed that such a classroom-based health promotion intervention in preschools resulted in a decrease in TV viewing by almost 25% in the intervention group, while the control group increased their TV viewing by almost 12%. However, no differences were observed in terms of body mass index (BMI) between the two groups [27]. The second review by Wahi et al. [25] additionally reported the study by Epstein et al. [28]. This study aimed to reduce TV viewing and computer use in 4- to 7-year-old preschoolers with a BMI ≥ 75th BMI percentile by using the device ‘the TV Allowance’. Children in the intervention group received a weekly time budget that could be spent on the TV Allowance to watch TV or play on the computer while the control group had free access to TV and computer. The results of this intervention showed that children in the intervention group reduced their TV viewing and computer use more compared to children in the control group [28]. Although a number of childhood overweight prevention interventions in different age groups (e.g., HELENA, IDEFICS, ENERGY) has been conducted targeting different energy-balanced related behaviors (e.g., physical activity, dietary intake), an intervention targeting different forms of sedentary behaviors (not only screen viewing behaviors but also the interruption of prolonged periods of sitting down) in preschoolers could not be identified. The ToyBox-study [29] aimed to develop and evaluate a 6-month intervention to prevent overweight in 4- to 6-year olds in six European countries (Belgium, Bulgaria, Germany, Greece, Spain and Poland). This study focused on increasing preschoolers’ water consumption, increasing the consumption of healthy snacks, increasing daily physical activity levels and decreasing different forms of sedentary behavior. The development and the implementation of the ToyBox-intervention included four different intervention modules addressing the four different behaviors mentioned above. For the development of the ToyBox-study intervention, the Intervention Mapping Protocol (IMP) [30] was used to ensure that the development was done on a scientific and systematic basis. The current paper will only focus on the sedentary behavior intervention module and aims to provide information on how the different steps of the IMP were implemented, and to present the matrices developed. This will help planners of future interventions aiming to decrease sedentary behavior in preschoolers to develop their own modules and matrices.

## Methods

**Figure 1:**
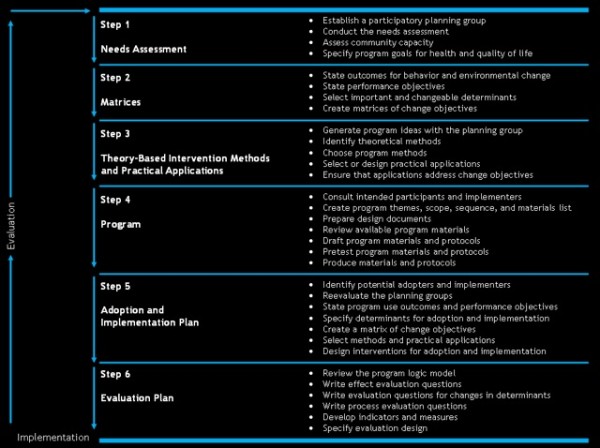
Overview of the Intervention Mapping steps and corresponding tasks.

The different steps included in the IMP are: 1) needs assessment, 2) preparing matrices of change objectives, 3) selecting theory-informed intervention methods and practical strategies, 4) producing intervention components and materials, 5) planning program adoption and implementation, and 6) evaluation planning (Figure 1) [30] (p.19). Two out of the six countries in this European study (Germany and Belgium) were responsible for the development of the different intervention modules, as described in the project’s description of work. Other participating countries provided feedback on the developed intervention and approved the intervention components and materials.

### Step 1: needs assessment

**Figure 2:**
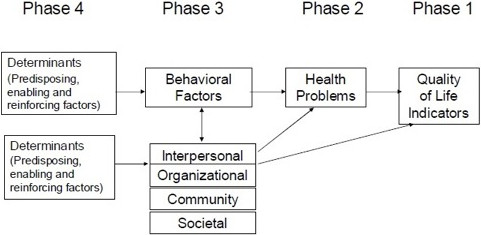
Overview of the PRECEDE model.

In the first step of the IMP, a needs assessment was executed to better understand the health problem of overweight in preschoolers and its association with sedentary behavior using the PRECEDE component [31] of the PRECEDE-PROCEDE model (Figure 2) [30] (p.36-37). This educational and ecological model in health program planning includes a sequence of steps and provides a detailed and well-structured approach for assessment procedures. During the first and the second phase of the PRECEDE component, quality of life indicators were investigated and a description of the health problem was formulated. Furthermore, because little is known about the link between sedentary behavior and overweight in preschoolers, the association between this behavior and overweight at the individual level was identified during the third phase of the PRECEDE model. Next to the behavioral analysis, an environmental analysis was performed, including the environmental factors at the interpersonal and organizational level that influence the health problem directly or through its behavioral causes. Because both parents/caregivers and preschool teachers have an important role in establishing behaviors, focus groups were executed at the interpersonal level to identify the influencing factors of preschoolers’ sedentary behavior, to identify possible difficulties and barriers to decrease this behavior and to get a better insight into the personal determinants of both the parents/caregivers and the teachers. Furthermore, the most important predisposing (preferences and habits of preschoolers), reinforcing (behaviors, knowledge and attitudes of parents/caregivers and teachers) and enabling (rules, barriers) factors of preschoolers’ sedentary behavior were collected during the last phase of the PRECEDE model [29]. The recruitment setting was country dependent, based on the country regulations and legislations. Specifically, in Germany, Bulgaria, Spain and Poland, recruitment was done in kindergartens, in Greece in kindergartens and in daycare centers and in Belgium in preschool settings. In order to ease reading, all these settings will be referred to as ‘kindergartens’ in this paper.

### Step 2: preparing matrices of change objectives

In the second step, a program goal for the health outcome to be achieved by the intervention was formulated. During this step of the IMP, the logic model of change was used to state the behavioral and environmental changes in terms of what is necessary to achieve the health outcome [30] (p.37). First, performance objectives were formulated to obtain behavioral and environmental outcomes that the intervention seeks to accomplish. These performance objectives state what the intervention participants have to do or how the environment has to be modified in order to achieve the health outcome. Based on the information derived from the focus groups and based on a literature search, determinants of behavioral and environmental outcomes were examined and listed. The list of potential determinants was then refined into a final determinant list by making a selection of each determinant in terms of relevance (strength of the association with sedentary behavior) and changeability (likelihood that the intervention could influence a change in the determinant). Finally, the selected determinants were crossed with the formulated performance objectives to generate matrices with change objectives. The change objectives were entered into cells formed at the intersection of each performance objective and determinant. These change objectives specify what needs to change in the determinants of behavioral or environmental outcomes in order to accomplish the performance objectives.

### Step 3: selecting theory-informed intervention methods and practical strategies

In the third step of the IMP, theoretical methods that can influence changes in the selected determinants were identified, based on a list including all change objectives by determinant. All change objectives that had to do with a specific determinant (e.g., knowledge) were listed and theoretical methods (e.g., active learning) were then matched with the corresponding determinant, resulting in a list including all change objectives by determinant with respective methods. Once the theoretical methods were selected, they were then translated into creative applications. The development of creative applications was guided by the formulated change objectives in the previous step of the IMP, and was also based on suggestions made by the teachers and the parents/caregivers during the focus groups.

### Step 4: producing intervention components and materials

In the fourth step of the intervention, the scope, the sequence, the themes and a list of necessary intervention materials were made. Suggestions made by the parents/caregivers and the teachers were used to prepare design documents for the production of the intervention materials that met the program objective. These design documents included a detailed description of the audience (i.e., preschoolers) and the contexts in which the material will be used. Once the design documents were developed, a review of available materials was done against the matrices and a list of methods and applications. Once initial design documents were created, culturally relevant program materials were developed.

### Step 5: planning program adoption and implementation

During the fifth step of the IMP, the intervention adoption and implementation was planned. Based on the first step of the IMP, teachers and parents/caregivers were chosen as intervention adopters and implementers, and local differences between and within the participating European countries were considered. Next, a clear implementation plan was developed to inform the researchers in the participating countries about the different core components of the intervention and about details of the steps in delivering the intervention. The emphasis of the implementation plan was placed on achieving a high level of fidelity and completeness, with the inclusion of implementation options to make the intervention more flexible for easy adoption.

### Step 6: evaluation planning

The last step of the IMP included the development of a plan for the evaluation of the outcomes and the process of the intervention. Next to the outcome evaluation, a process evaluation was performed and indicated the fidelity of the intervention delivery across the different countries. The last part of the sixth step in the IMP included the evaluation of the efficacy of the program in terms of costs and effects.

## Results

All the different steps of the IMP will be described briefly below, but a more detailed description will be provided for step 2 to step 4.

### Step 1: needs assessment

As part of the needs assessment, secondary data analyses were first executed between June 2010 and August 2010, indicating that the prevalence of overweight and obesity ranged from 8% to 30% and from 1% to 13% respectively in preschoolers between 4- and 7-years-old across the participating European countries [32]. A literature search was started in July 2010 and found moderate evidence for a positive association between TV viewing and overweight in preschool-aged children [33]. Results of the focus groups with European parents/caregivers executed between October 2010 and January 2011 indicated that preschoolers tend to like watching TV, with most parents/caregivers not expressing worries about this behavior. The most important influencing factors of preschoolers’ screen time were the weather conditions and parental habits at home [34]. According to the teachers, preschoolers do not sit very much at kindergarten. The lack of play space and play equipment were perceived as potentially influencing factors of preschoolers’ sedentary behavior during kindergarten hours [35].

### Step 2: preparing matrices of change objectives

Based on the information out of the first step of the IMP, three program objectives for the sedentary behavior intervention were formulated. The program objective for the individual level of the intervention was: *‘Children between 4- and 6-years-old decrease their sitting time (screen viewing activities and other sedentary activities) by 10*% *at home and during their time at kindergarten at the end of the intervention’*. Based on the program objectives, different performance objectives were formulated at the individual level of the child and for the parents/caregivers and teachers at the interpersonal and organizational level. An overview of the program objectives and performance objectives for each level of the intervention can be found in Table 1.

**Table 1:** Overview of the formulated program and performance objectives for each level of the intervention

**Level of the intervention**	**Target group**	**Program objective**	**Performance objective**
Individual level	Preschool child	Children between 4- and 6-years-old decrease their sitting time (screen viewing activities and other sedentary activities) by 10% at home and during their time at kindergarten at the end of the intervention	PO.1. Children decrease their total sitting time per day at kindergarten.
			PO.2. Children decrease their total sitting time per day at home or during leisure time.
			PO.3. Children limit screen viewing to one hour per day at kindergarten.
			PO.4. Children limit screen viewing to one hour per day or less at home (with help from their parents/caregivers).
			PO.5. Children switch from sitting down to standing up for some activities.
Interpersonal level	Parents/caregivers of preschoolers in the home environment	Parents/caregivers decrease their child’s sedentary time by 10% and limit screen viewing activities at home to less than one hour per day after the intervention.	PO.1. Parents/caregivers limit preschoolers’ screen viewing activities to one hour per day.
			PO.2. Parents/caregivers motivate (verbally) their children to do other activities instead of screen viewing activities.
			PO.3. Parents/caregivers do other activities together with their child instead of screen viewing activities.
			PO.4. Parents/caregivers are a role model for their children and limit their own time sitting down.
Organizational level	Teachers in the school environment	Teachers decrease preschoolers’ sedentary time by 10% at kindergarten and limit screen viewing activities to less than one hour daily after the intervention.	PO.1. Teachers use different strategies (e.g., classroom environmental changes, performing standing classroom activities, etc.) to decrease preschoolers’ total sitting time per day at kindergarten.
			PO.2. Teachers give assignments that the preschoolers need to fulfill standing up.
			PO.3. Teachers encourage the preschoolers to stand up when they are sitting down at the playground.
			PO.4. Teachers are a role model for the preschoolers and limit sitting down themselves.
			PO.5. Teachers encourage the preschoolers to switch from sitting down to standing up.

After all performance objectives were formulated for the individual, the interpersonal and the organizational level separately, personal determinants of sedentary behavior were listed for each level based on the results of the focus groups and a literature search. The selection of the determinants was based on their changeability, importance and strength of relationship with preschoolers’ behavior. The personal determinants selected for preschoolers were (i) attitude, (ii) preference, (iii) self-efficacy and (iv) capability. For parents/caregivers at the interpersonal level, the personal determinants included (i) self-efficacy, (ii) knowledge, (iii) attitude, (iv) habit and (v) social influence. Finally, for kindergarten teachers at the organizational level, (i) self-efficacy, (ii) habit, (iii) knowledge, (iv) attitude, and (v) social influence were selected as personal determinants. Once the personal determinants were listed, matrices of change objectives were created by crossing performance objectives with the selected determinants between January 2011 and the end of April 2011. Because performance objectives were formulated for each level of the intervention separately, a separate matrix was constructed for each level of the intervention (Table 2, 3 and 4). For example, the performance objective at the individual level for preschoolers to decrease their total sitting time per day at kindergarten was crossed with the determinant ‘preference’ and resulted in the change objective ‘*Children prefer to stand up instead of sitting down in the classroom or at kindergarten’*. The formulated change objectives were stated with an action word [30] (p.293), followed by a statement of what is expected to result from the intervention.

**Table 2:** Matrix for preschoolers at the individual level of the ToyBox-study intervention

**Performance objectives**	**Personal determinants**			
	**Attitude**	**Preference**	**Self-efficacy**	**Capability**
(**Preschoolers**)				
PO.1. Children decrease their total sitting time per day at kindergarten.	A.1. Children express positive feelings towards devoting less time sitting down at kindergarten.	P.1. Children prefer to stand up instead of sitting down in the classroom or at kindergarten.	SE.1. Children express confidence about decreasing their total sitting time per day at kindergarten, even when the other children want to sit down/are sitting down.	C.1. Children are capable of decreasing their total sitting time per day at kindergarten.
PO.2. Children decrease their total sitting time per day at home or during leisure time.	A.2. Children express positive feelings towards being less sedentary, at home or during leisure time.	P.2. Children prefer to stand up instead of sitting down at home or during leisure time.	SE.2. Children express confidence about decreasing their total sitting time per day at home or during leisure time, even when their siblings are sitting down.	C.2. Children are capable of decreasing their total sitting time per day at home or during leisure time.
PO.3. Children limit screen viewing to one hour per day at kindergarten.	A.3. Children express positive feelings towards limiting their screen viewing time by doing other non-sedentary activities at kindergarten.	P.3. Children indicate that they prefer to limit their screen viewing (e.g., TV viewing time, computer time, etc.) to less than one hour per day at kindergarten.	SE.3. Children express confidence about limiting their screen viewing to less than one hour per day at kindergarten, even when the other children are watching TV, playing on the computer or doing other screen viewing activities.	C.3. Children are capable of limiting their screen viewing time to less than one hour per day at preschool.
PO.4. Children limit screen viewing to one hour per day or less at home (with help from their parents/caregivers).	A.4. Children express positive feelings towards limiting their screen viewing time (with help from their parents/caregivers) by doing other activities at home.	P.4. Children prefer to limit their screen viewing (e.g., TV viewing time, computer time, etc.) to one hour per day or less at home (with help from their parents/caregivers).	SE.4. Children express confidence about limiting their screen viewing time to one hour per day (with help from their parents/caregivers) or less at home, even when their siblings are watching TV, playing on the computer or doing other screen viewing activities.	C.4. Children are capable of limiting their screen viewing time to one hour per day or less at home (with help from their parents/caregivers).
PO.5. Children switch from sitting down to standing up for some activities.	A.5. Children express positive feeling towards switching from sitting down to standing up for some activities.	P.5. Children prefer to switch from sitting down to standing up for some activities.	SE.5.a. Children express confidence about switching from sitting down to standing up, even when the other children do these activities sitting down.	C.5. Children are capable of switching from sitting down to standing up for some activities.
			SE.5.b. Children express confidence about switching from sitting down to standing up, even when the teacher does not give prompts to do this.	

PO.: performance objective.

Behavior: Decrease preschoolers’ sedentary behavior; Program objective: Children between 4- and 6-years old decrease their sitting time (screen viewing activities and other sedentary activities) by 10% at home and during their time at kindergarten at the end of the intervention.

**Table 3:** Matrix for parents/caregivers at the interpersonal level of the ToyBox-study intervention

**Performance objectives (Parents/caregivers)**	**Personal determinants**				
	**Self-efficacy**	**Knowledge**	**Attitude**	**Habit**	**Social influence**
PO.1. Parents/caregivers limit preschoolers’ screen viewing activities to one hour per day.	SE.1.a. Parents/caregivers express confidence that they can use different strategies to limit screen viewing activities even when their child wants to continue doing screen viewing activities.	K.1.a. Parents/caregivers know that it is recommended to limit screen activities of their child to one hour per day.	A.1. Parents/caregivers express positive feelings about the benefits that limiting the screen viewing activities one hour per day by using different strategies has for their child.	H1.a. Parents/caregivers plan no or only a limited amount of screen viewing activities of their child in their daily routine.	
	SE.1.b. Parents/caregivers express confidence that they can use different strategies to limit screen viewing activities even when their child is nagging.	K.1.b. Parents/caregivers list different strategies to limit screen viewing activities.		H.1.b. Parents/caregivers only turn on the TV after a certain time or at a certain moment (e.g., at 7 pm).	
				H.1.c. Parents/caregivers turn off the TV after a certain program or show or after a certain time.	
PO.2. Parents/caregivers motivate (verbally) their children to do other activities instead of screen viewing activities.	SE.2. Parents/caregivers express confidence that they can motivate their child to do other activities instead of screen viewing activities even when their child is nagging.	K.2. Parents/caregivers know how to motivate their child to do other activities instead of screen viewing activities (e.g., tips, tricks).	A.2. Parents/caregivers express positive feelings about the benefits that doing other activities instead of screen viewing activities has for their child.		SI.2. Parents/caregivers indicate that they are able to motivate their child to do other activities instead of screen viewing activities even when their colleagues/ friends/ neighbours don’t motivate their own child.
PO.3. Parents/caregivers do other activities together with their child instead of screen viewing activities.	SE.3. Parents/caregivers express confidence that they can do activities with their child which are not screen viewing activities even when their child only wants to do screen viewing activities.	K.3. Parents/caregivers list activities that can be done instead of screen viewing activities (e.g., tips, tricks, …).	A.3. Parents/caregivers express positive feelings about the benefits that doing other activities together with their child instead of screen viewing activities has for their child.	H.3. Parents/caregivers plan to do other activities together with their child before they turn on the TV at a certain time.	
PO.4. Parents/caregivers are a role model for their children and limit their own time sitting down.	SE.4.a. Parents/caregivers express confidence that they can be a role model for their child, even when they had a rough day.	K.4. Parents/caregivers know that being a role model for their child and limit their own time sitting down, encourages their child to also limit his/her time sitting down because they will copy this behavior.	A.4. Parents/caregivers express positive feelings about the benefits of being a role model for their children has for their child because the child will copy this behavior and therefore limit the time sitting down.	H.4. Parents/caregivers change their own habit and turn of the TV if they are together with their child.	
	SE.4.b. Parents/caregivers express confidence that they can be a role model for their child, even when they are tired or they are not in the mood.				
	SE.4.c. Parents/caregivers express confidence that they can be a role model for their child.				

PO.: performance objective.

Behavior: Decrease preschoolers’ sedentary behavior; Program objective: Parents/caregivers decrease their child’s sedentary time by 10% and limit screen viewing activities at home to less than one hour per day after the intervention.

**Table 4:** Matrix for teachers at the organizational level of the ToyBox-study intervention

**Performance objectives (teachers)**	**Personal determinants**				
	**Self-efficacy**	**Habit**	**Knowledge**	**Attitude**	**Social influence**
PO.1. Teachers use different strategies (e.g., classroom environmental changes, performing standing classroom activities, etc.) to decrease preschoolers’ total sitting time per day in kindergarten.	SE.1.a. Teachers express confidence that they can use different strategies to decrease preschoolers’ total sitting time in kindergarten even when they have a tight schedule to follow.	H.1. Teachers plan to use different strategies to decrease preschoolers’ total sitting time into their daily routine.	K.1.a. Teachers list different strategies to decrease preschoolers’ total sitting time a day.	A.1. Teachers express positive feelings about the benefits of using different strategies to decrease preschoolers’ total sitting time.	SI.1. Teachers indicate that they use different strategies to decrease preschoolers’ total sitting time per day in kindergarten even when other teachers stick to sedentary activities.
	SE.1.b. Teachers express confidence that they can use different strategies to decrease preschoolers’ total sitting time in kindergarten even when the preschoolers are lively.		K.1.b. Teachers know different strategies to decrease preschoolers’ total sitting time per day in kindergarten.		
PO.2. Teachers give assignments that the preschoolers need to fulfil standing up.	SE.2. Teachers express confidence that they can give assignments that the preschoolers need to fulfil standing up even when they need to rearrange their classroom.	H.2. Teachers plan to give assignments that the preschoolers need to fulfill standing up.	K.2. Teachers know assignments that the preschoolers need to fulfill standing up.	A.2. Teachers express positive feelings about the benefits of giving assignments that preschoolers need to fulfill standing up.	SI.2. Teachers indicate that they plan to give assignments that the preschoolers need to fulfill standing up even when other teachers stick to fulfilling assignments sitting down.
PO.3. Teachers encourage the preschoolers to stand up when they are sitting down at the playground.	SE.3. Teachers express confidence that they can encourage the preschoolers to stand up at the playground even when there is already a lot of noise and commotion.	H.3. Teachers plan to encourage the preschoolers to stand up at the playground every time they see the preschoolers sitting down.		A.3. Teachers express positive feelings about the benefits that encouraging the preschoolers to stand up at the playground has for the preschoolers.	SI.3. Teachers encourage the preschoolers to stand up when they are sitting down at the playground even when other teachers don’t do this.
PO.4. Teachers are a role model for the preschoolers and limit sitting down themselves.	SE.4. Teachers express confidence that they are a role model for the preschoolers in limiting sitting down even when they are tired.		K.4. Teachers know that being a role model for the preschoolers encourages the preschoolers to sit down less.	A.4. Teachers express positive feelings about being a role model for the preschoolers by limiting their own time sitting down.	
PO.5. Teachers encourage the preschoolers to switch from sitting down to standing up.	SE.5. Teachers express confidence that they can encourage the preschoolers to switch from sitting down to standing up, even when they don’t have the appropriate tools.	H.5. Teachers plan to encourage the preschoolers to switch from sitting down to standing up into their daily routine.	K.5. Teachers know that switching from sitting down to standing up is beneficiary for the preschoolers.	A.5. Teachers express positive feeling about the benefits of encouraging the preschoolers to switch from sitting down to standing up.	

PO.: performance objective.

Behavior: Decrease preschoolers’ sedentary behavior; Program objective: Teachers decrease preschoolers’ sedentary time by 10% at preschool and limit screen viewing activities to less than one hour daily after the intervention.

### Step 3: selecting theory-informed intervention methods and practical strategies

Methods from theory and from the literature that are capable of influencing changes in the determinants were chosen during the third step of the IMP. A systematic review that was conducted as part of the ToyBox-study identified effective behavioral models, methods and behavior change strategies that could be used in preschool children [36]. First, all determinants included in the matrices at different intervention levels were listed and were matched with methods derived from a theory. These methods were carefully considered for use in our intervention. For example, the formulated change objective *‘Children express positive feelings towards switching from sitting down to standing up for some activities’* was the result of crossing the performance objective *‘Switching from sitting down to standing up for some activities*’ with the determinant *‘attitude’*. The selected theoretical method that corresponded with the determinant ‘attitude’ in order to reach the change objective was ‘direct experience’ [30] (p.338). After the theoretical method was chosen, theoretical parameters and characteristics of the context were checked and the selected method was translated into a creative application. For example, a creative application that was formulated by the method of ‘direct experience’ was to decrease preschoolers’ sedentary behavior by completing activities (e.g., coloring, painting) standing up, with preschoolers directly experiencing what it is to complete these activities while standing up. Furthermore, for the translation of the methods into applications, the suggestions that parents/caregivers and teachers formulated during the focus groups were used. For example, kindergarten teachers indicated that providing information about sedentary behavior to preschoolers can be done by telling the preschoolers a story about a character whose levels of daily sedentary behavior they would like to decrease. Table 5 provides an overview of all the methods and applications that were selected and used to achieve the change objectives for each level of the intervention.

**Table 5:** Theoretical methods and applications for achieving the change objectives at each level of the ToyBox-study intervention

**Level of the intervention**	**Determinant**	Change objective *	**Method †**	**Related theory ¥**	**Application**	**Evaluation methods or measures**
PRESCHOOLERS	Attitude	A.1., A.2., A.3., A.4., A.5.	Modeling	SCT	Stories of the kangaroo	Core questionnaire
		A.1., A.3., A.4., A.5.	Active learning	SCT	Classroom activities done at kindergarten listed in the handbook	Process evaluation
		A.1., A.2., A.3., A.4., A.5.	Direct experience	TL	Activities done while standing up (provided in the handbook and in the movement breaks)	Core questionnaire Process evaluation
					Classroom activities done at kindergarten listed in the handbook	Process evaluation
		A.1.	Repeated exposure	TL	Repeated interruption moments at kindergarten	Objectively measured sedentary time with the accelerometer
					Classroom activities done at kindergarten listed in the handbook	Process evaluation
		A.4.	Consciousness raising (providing information)	HBM	Stories of the kangaroo	Core questionnaire
	Preference	P.1., P.2., P.3.a., P.3.b., P.4., P.5.	Modeling	SCT	Stories of the kangaroo	Core questionnaire
		P.1., P.2., P.5.	Active learning	SCT	Classroom activities done at kindergarten listed in the handbook	Process evaluation
		P.1., P.2.,	Direct experience	TL	Activities done while standing up (provided in the handbook and in the movement breaks)	Core questionnaire Process evaluation
					Classroom activities done at kindergarten listed in the handbook	Process evaluation
	Self-efficacy	SE.1., SE.2., SE.3., SE.4., SE.5.a., SE.5.b.	Active learning	SCT	Classroom activities done at kindergarten listed in the handbook	Process evaluation
		SE.1., SE.2., SE.3., SE.4., SE.5.a., SE.5.b.	Direct experience	TL	Activities done while standing up (provided in the handbook and in the movement breaks)	Core questionnaire Process evaluation
					Classroom activities done at kindergarten listed in the handbook	Process evaluation
		SE.1., SE.2., SE.3., SE.4., SE.5.a., SE.5.b.	Modeling	SCT	Stories of the kangaroo	Core questionnaire
	Capability	C.1., C.2., C.5.	Active learning	SCT	Classroom activities done at kindergarten listed in the handbook	Process evaluation
		C.1., C.2., C.3., C.4., C.5.	Modeling	SCT	Stories of the kangaroo	Core questionnaire
		C.1., C.2.	Verbal persuasion	SCT	Stories of the kangaroo	Core questionnaire
					Classroom activities done at kindergarten listed in the handbook	Process evaluation
PARENTS/CAREGIVERS	Self-efficacy	SE.1.a, SE.1.b., SE.2., SE.3., SE.4.a., SE.4.b., SE.4.c.	Guided practice	SCT	Newsletters	Core questionnaire Process evaluation parents/caregivers
		SE.1.a., SE.1.b., SE.3., SE.4.a., SE.4.b., SE.4.c.	Modeling	SCT	Tip cards	Core questionnaire Process evaluation parents/caregivers
					Newsletters	Core questionnaire Process evaluation parents/caregivers
		SE.1.b, SE.2., SE.3.	Verbal persuasion	SCT	Tip cards	Core questionnaire Process evaluation parents/caregivers
					Newsletters	Core questionnaire Process evaluation parents/caregivers
					Poster	Core questionnaire Process evaluation parents/caregivers
		SE.1.b., SE.2.	Goal setting	GST	Newsletters	Core questionnaire Process evaluation parents/caregivers
					Tip cards	Core questionnaire Process evaluation parents/caregivers
		SE.2.	Consciousness raising (providing information)	HBM	Tip cards	Core questionnaire Process evaluation parents/caregivers
					Newsletters	Core questionnaire Process evaluation parents/caregivers
					Parent-child activities at kindergarten	Core questionnaire Process evaluation parents/caregivers
		SE.4.b., SE.4.c.	Discussion	TIP	Parent-child activities at kindergarten	Core questionnaire Process evaluation parents/caregivers
					Newsletters	Core questionnaire Process evaluation parents/caregivers
	Knowledge	K.1.a., K.1.b.	Active learning	SCT	Tip cards	Core questionnaire
		K.1.a., K.1.b., K.2., K.3.,	Consciousness raising (providing information)	HBM	Tip cards	Core questionnaire
					Newsletters	Core questionnaire
					Parent-child activities at kindergarten	Core questionnaire Process evaluation parents/caregivers
		K.2., K.4.	Discussion	TIP	Parent-child activities at kindergarten	Core questionnaire
		K.3., K.4.	Guided practice	SCT	Tip cards	Core questionnaire
					Newsletters	Core questionnaire
	Attitude	A.1., A.2., A.3., A.4.	Arguments	PCM	Newsletters	Core questionnaire
		A.1., A.2., A.4.	Self-reevaluation	SCT	Newsletters	Core questionnaire
		A.2., A.3., A.4.	Discussion	TIP	Newsletters	Core questionnaire
					Parent-child activities at kindergarten	Core questionnaire
	Habit	H.1.a., H.3.	Guided practice	SCT	Newsletters	Core questionnaire
					Poster	Core questionnaire
		H.1.a., H.1.b., H.3.	Modeling	SCT	Newsletters	Core questionnaire
					Tip cards	Core questionnaire
	Social influence	SI.2.	Resistance to social pressure	TPB	Newsletters Tip cards	Core questionnaire
TEACHERS	Self-efficacy	SE.1.a., SE.1.b., SE.2., SE.3., SE.4., SE.5.	Guided practice	SCT	Teachers’ guide	Teachers’ questionnaire Process evaluation teachers
					Teachers’ training	Process evaluation teachers
					Classroom activities done at kindergarten listed in the handbook	Teachers’ questionnaire Process evaluation teachers
		SE.1.a., SE.1.b., SE.2.	Consciousness raising (providing information)	HBM	Teachers’ training	Process evaluation teachers
					Teachers’ guide	Process evaluation teachers
					Stories of the kangaroo	Teachers’ questionnaire Process evaluation teachers
					Classroom activities done at kindergarten listed in the handbook	Teachers’ questionnaire Process evaluation teachers
		SE.1.b., SE.2., SE.3., SE.4., SE.5.	Modeling	SCT	Teachers’ guide	Teachers’ questionnaire Process evaluation teachers
					Teachers’ training	Process evaluation teachers
		SE.1.b., SE.2., SE.4., SE.5.	Discussion	TIP	Teachers’ training	Process evaluation teachers
	Habit	H.2., H.3., H.4.	Modeling	SCT	Teachers’ training	Process evaluation teachers
					Teachers’ guide	Teachers’ questionnaire Process evaluation teachers
		H.2., H.3., H.4.	Planning coping responses	TGDB	Teachers’ training	Process evaluation teachers
					Teachers’ guide	Teachers’ questionnaire Process evaluation teachers
	Knowledge	K.1.a., K.1.b., K.2., K.4., K.5.	Consciousness raising (providing information)	HBM	Teachers’ training	Teachers’ questionnaire Process evaluation teachers
					Teacher’s guide	Teachers’ questionnaire Process evaluation teachers
					Stories of the kangaroo	Teachers’ questionnaire
		K.1.a., K.1.b., K.2., K.4., K.5.	Discussion	TIP	Teachers’ training Parent-child activities at kindergarten	Teachers’ questionnaire Process evaluation teachers
	Attitude	A.3., A.4.	Consciousness raising (providing information)	HBM	Teachers’ training	Teachers’ questionnaire Process evaluation teachers
					Teachers’ guide	Teachers’ questionnaire Process evaluation teachers
		A.3., A.4.	Discussion	TIP	Teachers’ training	Teachers’ questionnaire Process evaluation teachers
	Social influence	SI.2., SI.3.	Resistance to social pressure	TPB	Teachers’ training	Teachers’ questionnaire Process evaluation teachers
					Teachers’ guide	Teachers’ questionnaire Process evaluation teachers

*See Table 2, 3 and 4 for a full description of the change objectives.

† A description of the methods mentioned in Table 5 can be found Bartholomew et al. 2011, page 322, Table 6.4 until 6.16.

*¥* SCT: Social Cognitive Theory; TIP: Theories of Information Processing; PCM: Persuasion-Communication Matrix; TL: Theories of Learning; TPB: Theory of Planned Behavior; GST: Goal-Setting Theory; TGDB: Theories of Goal Directed Behavior; HBM: Health Belief Model.

### Step 4: producing intervention components and materials

Based on the selected methods and applications, a comprehensive intervention program and a list of intervention materials were compiled. The program materials for the individual level and the organizational level included a general teachers’ guide and a hand book with classroom activities. In the teachers’ guide, some general information was provided (e.g., a definition of sedentary behavior, prevalence of preschoolers’ sedentary behavior). The handbook consisted of three different parts. First, environmental changes that teachers could perform in their classroom were suggested. For example, teachers could put the sandbox on top of a table so that the preschoolers have to stand up to play with the sand in the sandbox instead of sitting down. Secondly, fun movement breaks were provided for the teachers to implement during the day or on days when no physical education classes were carried out at kindergarten (e.g., asking the preschoolers to walk around in the classroom and stand still when a certain word is said). Kindergarten teachers were encouraged to set time rules for interrupting preschoolers’ sedentary behavior during the school hours (e.g., every 30–40 minutes per day) and to execute two movement breaks in the morning and two in the afternoon. Finally, the third part of the handbook included three stories about a kangaroo and his friends. Teachers could read these stories to increase preschoolers’ knowledge, enhance preschoolers’ skills and their self-efficacy. Implementing activities of the sedentary behavior intervention for a minimum of one hour per week was recommended.

Because parents are perceived as children’s most important caregivers [37], the home environment at the interpersonal level was also included in the intervention. In order to involve the parents/caregivers in this kindergarten-based intervention, different educational materials were developed: a poster (to be colored by the preschoolers), two newsletters and two tip cards. Teachers were asked to hand out these materials to the parents/caregivers according to the provided time plan. In week 13 of the intervention, the poster and newsletter 1 were handed out. Following this, the first tip card on sedentary behavior was handed out in week 15. During the repetition period, the second newsletter and the second tip card were handed out in week 23 and week 24 respectively. The poster included key messages to decrease preschoolers’ sedentary behavior at home (e.g., ‘Don’t sit down too long – stand up and be active’), newsletters included information about sedentary behavior and in the tip cards, different tips and strategies to decrease this behavior were mentioned. Furthermore, activities that parents/caregivers and children could perform together at home to decrease sedentary behavior were also suggested in the educational materials. Table 6 provides a detailed overview of what was included in the different intervention materials. After the end of the ToyBox-study (April 2014), all the different intervention materials will be available on the website (http://www.toybox-study.eu).

**Table 6:** Detailed description of the different applications used in the intervention

**Individual level and organizational level**^**a**^	Handbook: Stories of the kangaroo and its friends (a cat, a bear and a rabbit)	1. Story 1: the kangaroo and his friends start an adventurous trip to the forest in search of a cave. The cat prefers to watch TV instead and the other animals wanting to change the cat’s behavior, try to persuade her that a real adventure is much more fun compared to watching TV.
		2. Story 2: the animals arrive at school only to discover that their teacher, Mrs. Owl, has mysteriously disappeared. They have to choose whether they will watch TV or start a new adventure and search for their favorite teacher.
		3. Story 3: the kangaroo received a new present; a pair of magic socks that stopped him from being sedentary. But in the end, the little kangaroo understands that it is not magic that makes him move; it is actually what his own body is asking for.
	Handbook: Classroom activities in the form of short and long movement breaks	Short (1–5 minutes) movement breaks: e.g., ‘Playing a statue’: preschoolers have to walk through the classroom and when the teachers says ‘STOP’, they have to stay still for some seconds.
		Long (15–20 minutes) movement breaks: e.g., preschoolers can walk through the classroom and when the teachers says ‘SEARCH A FRIEND’, each preschooler have to look for a friend to stand still for about 3 seconds.
	Activities to be done while standing up (provided in the handbook and in the movement breaks)	Different activities were listed. For example:
		- The teacher can remove the chairs and let the preschoolers paint or color on a raised desk
		- Putting the sandbox on a raised desk for the preschoolers to play while standing up.
	Repeated interruption moments at kindergarten	Kindergarten teachers could include repeated sitting interruption moments while preschoolers are at kindergarten. It is suggested in the handbook to interrupt prolonged periods of sitting down and set time rules to interrupt this behavior every 30 – 40 minutes.
	Parent–child activities at kindergarten	Parents/caregivers are invited to participate in these parent–child activities and to come to the kindergarten where they can do nice activities together with their child (e.g., a role play).
	Teachers’ guide	The teachers’ guide is developed for teachers and included the following topics:
		- An explanation of why we need the ToyBox-study
		- A description of all the different materials that are included in the ToyBox-study and how these materials should be used
		- Some information on how they can be a team together with the parents/caregivers to change different preschoolers’ behaviors.
	Teachers’ training	All teachers from the intervention schools were invited to attend two different training sessions.
		1. First training session (June 2012): a general introduction was given to the teachers, the teachers’ guide was presented and the environmental changes teachers could perform in their classroom to change the different behaviors included in the ToyBox-study and the different classroom activities were explained. At the end of the training, there was also some time for discussion.
		2. Second training session (September – October 2012): teachers could first share experiences and a small repetition of the information provided in the first training was done. Afterwards, the teachers went through the classroom activities together with the researchers. The second training was closed by a discussion.
**Interpersonal level**^**b**^	Newsletters	A clear definition of sedentary behavior is included in the first newsletters and also the activities that cover this behavior are mentioned. Furthermore, also the recommendations for preschoolers’ sedentary behavior and screen time are included in the newsletters.
	Tip cards	Parents/caregivers are provided with different tips of how to decrease preschoolers’ sedentary behavior (e.g., try to avoid that your child turns the TV on without your permission).
		In these tip cards, parent–child activities that could be performed at home were suggested as well. For example, preschoolers and parents/caregivers could decrease their sedentary time by doing things in the household together instead of watching television together.
	Poster	Four different key messages were mentioned:
		- ‘Don’t sit down for a long time, get up and be active’
		- ‘Include active movement breaks in the children’s daily lives’
		- ‘Limit screen viewing activities – make your own experiences’
		- ‘Don’t eat in front of screens’
		On the posters, different pictures of the kangaroo were provided for the preschoolers to color.

^**a**^Applied at kindergartens by teachers. Usually in the form of classroom activities with the participation of all preschoolers.

^**b**^Addressed to parents/caregivers and designed to inform and involve the family.

### Step 5: planning program adoption and implementation

The strategic implementation of the intervention was planned during step five of the IMP. The ToyBox-study intervention was implemented in a cluster randomized controlled trial intervention with a pre-test posttest design including 300 kindergartens. Each kindergarten was assigned either to the intervention or control condition. In each participating kindergarten, at least one teacher and 20 preschoolers per class were included. In total, more than 7000 preschoolers and their parents/caregivers were targeted. Teachers in the intervention kindergartens focused on different behaviors for 24 weeks from October 2012 until the end of March 2013 (school year 2012–2013) and executed the sedentary behavior intervention from week 13 to week 17 and planned a repetition of this focus for two weeks in week 23 and 24 later that school year. During two different training sessions that were organized in the participating countries, teachers of the intervention schools were informed about their role as the implementers of the intervention and about the specific time plan of the intervention. Furthermore, the handbook and the different materials were presented, role play was done and teachers could discuss with each other how they would implement the intervention in their classroom.

### Step 6: evaluation planning

In the final step of the IMP, a plan to evaluate the effectiveness of the evidence-based family-involved intervention targeting preschoolers was developed. After the 24 intervention weeks, the general effectiveness of the intervention was evaluated with regards to changes in anthropometric measures (BMI) and changes in behaviors related to the specific program objectives. For the sedentary behavior component of the ToyBox-study intervention, preschoolers’ sedentary time was objectively measured with accelerometers in one country (Belgium) because these devices were available in this country. These measures were used to investigate whether preschoolers’ sedentary time decreased by 10% at home and during the time spent at kindergarten during the day (program objective at the individual level). Furthermore, to investigate potential changes in screen viewing activities, parents/caregivers in all countries were asked to complete a questionnaire during baseline and posttest measurements about the amount of time their child spent watching TV, using the computer and performing quiet play. A new primary caregivers’ questionnaire (i.e., core questionnaire) was developed for the ToyBox-study and reliability of this questionnaire was investigated. Results of this test-retest reliability showed that the core questionnaire is a reliable tool to assess behaviors of preschool children participating in a kindergarten intervention (González-Gil et al., unpublished data). This core questionnaire was used to investigate whether parents/caregivers limited preschoolers’ screen viewing activities at home to less than one hour (program objective at the interpersonal level). To investigate potential changes in preschool teachers’ behavior at preschool, a teachers’ questionnaire was developed that investigated whether kindergarten teachers limited preschoolers’ screen viewing activities to less than one hour. This questionnaire was completed by teachers during baseline and posttest measurements and included specific questions about different strategies to interrupt prolonged periods of sitting, movement breaks, and possibilities to rearrange the classroom to decrease sedentary behavior, etc. before and after the intervention (program objective at the organizational level).

To evaluate the implementation of the intervention at home and in the kindergartens, process evaluation questionnaires were developed for the teachers and the parents/caregivers. This process evaluation identified the parental activities that parents/caregivers performed at home to decrease preschoolers’ sedentary behavior and the activities teachers performed at preschool. After all questionnaires were developed and approved by all partners, they were translated from English into local languages. After the translation to the local language, these questionnaires were back translated to detect any potential differences between the back translated and the original questionnaires. Finally, a health economic modeling model was used to analyze the cost effectiveness of the intervention. This model consisted of a decision analytic model to represent either the probability of improved healthy behavior or improved BMI category in preschoolers and of a Markov model simulating over a lifetime the occurrence of obesity-related complications with and without the early childhood intervention (Pil et al., unpublished data).

## Discussion

The present paper described the evidence-based development of an intervention, focusing on sedentary behavior. This sedentary behavior intervention was the first intervention that targeted different forms of sedentary behavior. A description of the different steps towards the development of the intervention using the IMP is provided, including detailed information about the preparation of the matrices with change objectives, the selection of methods and applications and about the production of the intervention materials. The development of this intervention matched the systematic evidence-based approach of the IMP and was perceived as rather complex and time-consuming. The matrices and change objectives were developed by two countries and were afterwards discussed during the ToyBox-study meeting that took place in Ghent (Belgium) during the first week of March 2011. After all partners approved the matrices and change objectives, the matrices were finalized by the end of April 2011. The second step of the IMP took approximately four months and was considered as an iterative process, because drafts of matrices and change objectives were read, discussed and approved by the participating countries. Other studies that used the IMP for the development of an intervention also mentioned that this process was time-consuming [38,39]. Planners of future health interventions should take into consideration that planning an intervention using the IMP requires scientific staff and necessary funding. Even when researchers want to develop an intervention that is less complex, has less intervention levels (e.g., only the kindergarten level) or focuses on less behaviors, it is recommended to pay enough attention to the selection of these intervention developers and to ensure that at least of few of the intervention developers have experience with using the IMP and developing matrices. Furthermore, it is important to devote enough time on the execution of the different IMP steps to ensure that well-considered choices are made in the development and implementation of the intervention. Although this process was experienced taking much time and effort, it is an effective approach to making choices systematically for the development and implementation of an intervention. By making the process of the development of our intervention more transparent, we hope to convince intervention developers of the added value of using the IMP for the development of health promotion interventions. The detailed description of the IMP process will also be an aid for intervention developers in order to avoid repetitive work for the design of similar interventions.

Going through the different steps of the IMP, it became clear that two important agents for targeting preschoolers’ sedentary behavior could be identified, namely the parents/caregivers and the teachers. The school setting provided an ideal opportunity to emphasize and to promote an active and healthy lifestyle in this age group [40] and to decrease preschoolers’ sedentary behavior. In the European countries participating in the ToyBox-study intervention, enrolment rates of preschoolers in preschool classes or kindergartens are particularly high (between 95 - 99%) [41]. Because of the high enrolment rate and because children are especially influenced by their family/parents and the kindergarten environment [42,43], the inclusion of both these target groups was of high importance to decrease preschoolers’ sedentary behavior. During the intervention, changing teachers’ behavior and changes in the classroom environment that could easily be performed by the kindergarten teachers were targeted, while changes in kindergarten’ policies were not included. Kindergarten’ policies are often formulated and defined by higher executive boards, what makes these policies much more difficult to change during an intervention compared to a classroom environment. However, striving for a policy to limit screen time at kindergarten or to use it only for educational purposes might be an important strategy that could be included in further intervention studies.

Different reviews mentioned that indirect methods were mostly used to engage parents/caregivers [44-46]. Furthermore, parents of older children (10–12 year olds) emphasized that their child’s behavior and not their own behavior should be targeted in interventions [47]. To ensure that parents/caregivers in our intervention did not feel like their own behavior was being targeted in particular, and that they were not overloaded with information, indirect methods were used to provide parents/caregivers with information about appropriate sedentary behavior in preschoolers on a regular base. So far, only one study that aimed to decrease TV viewing time in Australian primary school children [48] has already used newsletters to provide parents/caregivers with tips on how sedentary behavior can be changed. However, the effectiveness of this indirect strategy in preschooler parents/caregivers is yet unknown. According to the IMP, a planning group that includes stakeholders should be established before the start of the development of the intervention. Because the ToyBox-study included six different European countries, establishing a planning group including community members, program implementers, etc. from all participating countries was not possible. To ensure that a meaningful participation of European stakeholders was obtained, focus groups were executed with parents/caregivers and teachers as the potential program participants. Each country was also advised to have close contact with stakeholders and implementers so that they could address important issues before, during and after the development of the intervention. In the literature, focus groups with parents/caregivers and preschool teachers have been executed to describe the influencing factors, barriers and facilitators of preschoolers’ physical activity levels [49-52], but little information could be located about the most important predisposing, reinforcing and enabling factors of preschoolers’ sedentary behavior. Therefore, parents/caregivers and teachers were invited to discuss their opinions on preschoolers’ sedentary behavior. Although the execution of focus groups enabled us to collect primary data that could be used in the needs assessment to get a better insight into preschoolers’ sedentary behavior at home and in the kindergarten environment, a well-functioning planning or working group might be even more able to interpret the needs and perspective of preschoolers and the target agents of the intervention.

Once results of the focus groups from all countries in the ToyBox-study were analyzed, general recommendations for the intervention were formulated and taken into account during the development of the intervention with the IMP. As suggested in another multi-center prevention program with activities for health promotion (namely, the IDEFICS study), specific focus group results and suggestions were used during the local and cultural adaptation of the intervention component [38]. Because results of the focus groups indicated some cultural differences between the participating countries, the formulation of general recommendations for the ToyBox-study was well thought-out. Planners of future interventions including different European countries are advised to ensure that local and cultural adaptations of the intervention, based on cultural differences between the countries, are possible. For the sedentary behavior module of the intervention, cultural adaptations were rather limited, because of sedentary behavior being a new behavior to parents/caregivers and preschool teachers. So, apart from the adaptations that had to be done for other behaviors (e.g., the availability of fruit in preschoolers differed largely across the European countries), the intervention module focusing on sedentary behavior was quite similar in all participating countries.

Because some adaptations to cultural differences between the countries were needed in the intervention materials for some behaviors, pre-testing the material in all intervention countries, as part of the fourth step in the IMP, was not possible. However, future planners of health promotion interventions are advised to devote enough time on pretesting the materials and on discussing intervention materials with the implementers. The implementers of interventions can potentially address important issues and possible traps about the materials that intervention developers can easily adapt. For example, Belgian preschool teachers’ suggested that additional visual support (e.g., including illustrations in the kangaroo stories) in the stories would make it easier to read the stories to the preschoolers and would possibly transfer the information in a more successful way. By pre-testing the intervention materials, including successful theories of information processing [30] (p.443-448) and by adopting the materials in accordance with implementers’ feedback, the quality of the intervention and the materials will increase even more and this will possibly have a positive effect on the effectiveness of the intervention. The feedback on the intervention materials provided by the teachers during and after the implementation will be taken into account when the intervention material is revised after the study has ended.

Results from the literature indicated that studies conducted on preschoolers’ sedentary behavior mainly focused on decreasing preschoolers’ screen viewing activities [27,28] and changes in these behaviors were mainly measured. Results of the study by Dennison et al. [27] and Epstein et al. [28] indicated mainly changes in sedentary behavior measured by the amount of TV or video viewing, while in this intervention, changes in different forms of sedentary behaviors and changes in BMI were measured. To indicate possible effects of the intervention on time spent in screen viewing activities, subjective methods (i.e., a parental and a teachers’ questionnaire) were used. In addition, objective measures of preschoolers’ sedentary behavior were also assessed in one country (Belgium) to evaluate if the intervention was effective in decreasing preschoolers’ total sedentary time. Future planners are suggested to make well-considered choices of how they will measure intervention effects. We believe that objective measures, in conjunction with subjective measures should be used to assess both total sedentary time and preschoolers’ time spent in different sedentary activities.

## Conclusions

A systematic approach using the IMP was used for the development of the intervention part of the ToyBox-study, which among others aimed to reduce different forms of sedentary behavior in preschoolers in six European countries. The second IMP step was proven to be the most iterative and time-consuming of all, as the development of matrices was a rather meticulous task. The final outcome of this IMP exercise was a standardized intervention that was implemented in the different participating European countries, with some minor adaptations to account for some cultural differences. Despite the fact that implementing IMP was a rather long and systematic process, the evidence-based development of this intervention could increase its effectiveness. Planners of future interventions are encouraged to carefully select the developers and ensure that enough time and money are available for the preparation of an intervention. Future intervention developers can use the matrices as an aid in order to avoid repetitive work for the development of their intervention.

## Abbreviations

TV: Television; BMI: Body mass index; IMP: Intervention mapping protocol.

## Competing interests

The authors declare that they have no competing interests.

## Authors’ contributions

All authors participated in the ToyBox-study and in the study design. All authors read, critically reviewed the manuscript and approved the final manuscript. EDD, MDC, IDB and GC were responsible for the development of the sedentary behavior intervention of the ToyBox-study. EDD wrote the manuscript.
